# Synergistic Microbicidal Effect of Auranofin and Antibiotics Against Planktonic and Biofilm-Encased *S. aureus* and *E. faecalis*

**DOI:** 10.3389/fmicb.2019.02453

**Published:** 2019-10-24

**Authors:** Pengfei She, Linying Zhou, Shijia Li, Yiqing Liu, Lanlan Xu, Lihua Chen, Zhen Luo, Yong Wu

**Affiliations:** Department of Clinical Laboratory, The Third Xiangya Hospital of Central South University, Changsha, China

**Keywords:** auranofin, biofilm, combination therapy, subcutaneous abscess model, *Staphylococcus aureus*, *Enterococcus faecalis*

## Abstract

Methicillin-resistant/susceptible *Staphylococcus aureus* (MRSA/MSSA) and *Enterococcus faecalis* strains are often found in community- and hospital-acquired infections. The single use of conventional antibiotics hardly completely kills the bacterial cells of interest, especially in the form of biofilms. Thus, drug repurposing and antimicrobial combination are promising ways to solve this problem. Antimicrobial susceptibility assays against cocci in a suspension and in a biofilm mode of growth were performed with broth microdilution methods. Checkerboard assays and the cutaneous mouse infection model were used to examine the activity of auranofin and conventional antibiotics alone and in combination. In the present study, auranofin possesses potent antimicrobial activities against both planktonic cells and biofilms with minimum inhibitory concentrations ranging 0.125–0.5 mg/L. Auranofin in combination with linezolid or fosfomycin showed synergistic antimicrobial activities against *S. aureus* MSSA and MRSA both *in vitro* and *in vivo*. Similarly, auranofin also behaved synergistic effect with chloramphenicol against *E. faecalis.* Additionally, auranofin improved the antibiofilm efficacy of chloramphenicol and linezolid, even on the biofilms grown on a catheter surface. Though, *S. epidermidis* showed significant susceptibility to AF treatment, no synergistic antimicrobial effects were observed with antibiotics we tested. In all, the use of a combination of auranofin with linezolid, fosfomycin, and chloramphenicol can provide a synergistic microbicidal effect *in vitro* and *in vivo*, which rapidly enhances antimicrobial activity and may help prevent or delay the emergence of resistance.

## Introduction

*Staphylococcus aureus* and *Enterococcus faecalis* have been known to be responsible for most of healthcare- and nosocomial-associated infections. *S. aureus* could cause polymicrobial infections with many pathogens, such as enterococcus (Reyes et al., 2010; Hayakawa et al., 2013), *Pseudomonas aeruginosa* (Radlinski et al., 2017; Alves et al., 2018), *Peptostreptococcus anaerobius* (Yamagishi et al., 2017), *Streptococcus pyogenes* (Gilmer et al., 2013), and even Candida species (Nash et al., 2015; Todd et al., 2019), which are hard to be eradicated and finally led to a striking mortality rate. According to the report by the SENTRY antimicrobial surveillance program (North America), the main pathogens isolated from skin as well as soft tissue infections (SSTIs) now include 45.9% *S. aureus* and 8.2% *Enterococcus* sp. (Rennie et al., 2003). SSTIs abscesses, for instance, create fluid, pus-filled pockets infiltrated by bacteria as well as inflammatory cells, and are frequently extremely resilient to conventional antibiotic therapy (Ki and Rotstein, 2008). In addition, abscesses are the utmost common sign for high-dose, recurrent and long-term intravenous broad-spectrum antibiotic administration (Ramakrishnan et al., 2015).

Biofilms are a widespread problem in healthcare facilities and hospitals. Indeed, the United States National Institutes of Health reported that 80% of chronic infections are related to biofilms (Monroe, 2007). The attachment of *S. aureus*, *S. epidermidis*, and *E. faecalis* onto tissues or the surface of medical apparatuses contributes to the pathogenesis of infection (Archer, 1998). The bacterial cells living in a biofilm are responsible for a number of chronic infections and become resilient to antibiotics as well as host-defense mechanisms (Gomes et al., 2009).

Recently, many studies have been conducted to address the repurposing of FDA-approved drugs as new antimicrobial agents. Auranofin (AF) is a gold-containing compound and prescribed for the treatment of rheumatoid arthritis (Glennas et al., 1997). The study of AF for its antimicrobial effects and inhibition of biofilm formation is an attractive possible treatment approach (Natsis and Cohen, 2018). Researchers found its antimicrobial efficacy against cocci (including *Staphylococcus* sp. and *E. faecalis*) and *Mycobacterium tuberculosis*. AF employs its effects via a distinctive process comprising the prevention of TrxR, and it maintains action against current antibiotic-resistant strains (Cassetta et al., 2014; Harbut et al., 2015; Fuchs et al., 2016). In addition, AF compared with most of the conservative medications available might be an appropriate feature in the fight against a dynamic as well as quickly altering microbial community such as biofilms.

AF shows good antimicrobial effects on cocci and AF in combination with topical antibiotics (mupirocin, retapamulin, and fusidic acid) exhibits additive antimicrobial activity against MRSA (Thangamani et al., 2016). However, to the best of our knowledge, there is no research reporting combinationary therapy with conventional systemic administration associated antibiotics in a subcutaneous abscess infections model. In the present study, we showed the antimicrobial and antibiofilm activities of AF alone or in combination with conventional antibiotics against *S. aureus* and *E. faecalis* strains *in vitro* and *in vivo*.

## Materials and Methods

### Bacterial Strains

AF and antibiotics [fosfomycin (FOF); ciprofloxacin (CIP); tetracycline (TET); linezolid (LZD); chloramphenicol (CHL); levofloxacin (LVX); teicoplanin (TEC); clindamycin hydrochloride (CLI) hydro; gentamicin (GEN); vancomycin (VAN)] were purchased from the MedChemExpress company (Monmouth Junction, NJ, United States). *E. faecalis* ATCC 29212, *S. aureus* ATCC25923 and ATCC29213 were kindly provided by Juncai Luo (Tiandiren Biotech, Changsha, China). *S. epidermidis* RP62A and ATCC 12228 were given by Di Qu (Shanghai Medical College of Fudan University), *S. aureus* ATCC43300 (MRSA), Newman, and RJ-2 were given by Min Li (Renji Hospital, Shanghai Jiao Tong University School of Medicine). Other clinical strains were isolated from the wound secretion or sputum of inpatients at the Third Xiangya Hospital of Central South University. *Staphylococcus* spp. were grown in tryptic soy broth (TSB) broth medium (Solarbio, Shanghai, China), and *E. faecalis* was grown in brain heart infusion (BHI) broth medium (Solarbio, Shanghai, China) at 37°C.

### Susceptibility Testing of Planktonic Bacteria

Bacterial strains were cultured in cationic corrected Mueller–Hinton (MH) broth (BD/Difco, United States). Susceptibility tests were performed by twofold regular broth microdilution of the test compounds, as recommended by the Clinical and Laboratory Standards Institute (CLSI) (Harbut et al., 2015). After 16–18 h of incubation at 37°C, the nominal concentration necessary to stop the development of test bacteria was defined as the MIC, and the minimum bactericide concentration (MBC) was identified depending on the lowermost concentration of antimicrobials that killed 99.9% of the test bacteria by spreading the bacterial culture out onto a suitable agar plate (CLSI, 2005).

### Susceptibility Testing of Biofilms

For *S. aureus* biofilm determination. The culture was grown overnight in TSB and successively diluted 1:50 in TSB to achieve an absorbance at 630 nm of ∼0.1. Two hundred microliter aliquots of the diluted culture were added to every well of a microtiter plate and incubated at 37°C for 24 h. For *E. faecalis* biofilm determination, bacterial suspensions (18 μL) from overnight cultures were mixed with 162 μL of BHI in the wells, and biofilms were allowed to form on the plates for 24 h (Lee et al., 2012).

Following the incubation, the contents were removed and rinsed, 50 μL of medium and 50 μL of the specified drug were added to every well, and incubated at 37°C for 24 h. Then, the contents were removed and the remaining biofilms were determined by crystal violet (CV), XTT staining or live cell count as follows:

(1) CV staining (Holmberg et al., 2012). Each well was stained with 100 μL of 0.25% CV for 15 min. The wells were rinsed and dissolved with ethanol for 20 min. The absorbance was determined at 570 nm.
(2) XTT staining. One hundred microliters of a solution comprising 200 mg/L of XTT and 20 mg/L of phenazine methosulfate (MACKLIN, Shanghai, China) was mixed in each well, and the incubations were performed and incubated for 3 h at 37°C in the dark. The absorbance was determined at 490 nm (Nesse et al., 2015). The definition of MBEC30/MBEC50/MBEC70 were defined as the minimal concentration of the particular antimicrobial’s ability to inhibit 30/50/70% growth of the biofilms, respectively, compared to the control group (Gomes et al., 2009).
(3) Biofilm viable count (Mataraci and Dosler, 2012). One hundred microliters of 1 × PBS was aliquoted into each well, and the contents were scrapped and mixed thoroughly with pipette tips. A sample volume of 100 μL was plated onto blood agar and successively diluted with a saline solution before plating onto additional agar plates.

### Checkerboard Assays for Planktonic Bacteria

The impacts of individual antibiotics and in combination with AF were evaluated using the broth microdilution checkerboard technique (Odds, 2003; Flamm et al., 2019). Each microtiter well-comprising the designated combination of antibiotics was inoculated with an overnight culture diluted to provide an absolute concentration of ∼5 × 10^5^ CFU/ml. Following incubation, the optimal fractional inhibitory concentration index (FICI) was measured as the minimal inhibitory concentration of the combination divided by that of the single antibiotic (Odds, 2003): FICI ≤ 0.5 designates synergy; 0.5 < FICI ≤ 4.0 designates no interaction; FICI > 4.0 designates antagonism.

### Checkerboard Assays for Preformed Biofilms

The preparation of overnight biofilms was the same as explained earlier in this study. The biofilms were rinsed, twofold sequential dilutions of antibiotics and AF in a 96-well microtiter plate were prepared, and 100 μL of these mixtures were added to the biofilms. Concentration ranges, as recognized with susceptibility testing, were utilized for the antibiotics as well as the AF. Following an incubation for 24 h at 37°C, the medium containing antimicrobials was removed, and 100 μL of XTT with PSM was added as described above (Koppen et al., 2019). The MBEC50 values were quantified.

### Antibiofilm Effect of AF on Catheters

To study the efficacy of AF combined with antibiotics against biofilms on catheters, overnight cultures of the biofilm-forming strains were diluted 1:40 in TSB (*S. aureus*) or BHI (*E. faecalis*) containing 5% rabbit plasma. Catheter (Jerry infusion set, Shandong, China) pieces (1 cm in size) were cut, divided into two halves, and added to the culture. Next, they were incubated at 37°C for 24 h. Afterward, the catheters were removed and washed. The biofilms on catheters were challenged with AF alone or in combination with antibiotics for 24 h. The catheters were scratched by an inoculation loop and sonicated for 15 min. Then, the samples were vortexed carefully and plated on blood agar plates (Nair et al., 2016).

### Confocal Laser Scanning Microscopy (CLSM)

The above-treated bacteria were cultured on glass cover slides and incubated with 10 μL of 1000-fold diluted SYTO9 fluorescent staining solution and propidium iodide at a ratio of 1:1 (vol/vol) for 15 min in the dark. After rinsing, the stained biofilm was examined with a CLSM (Zeiss LSM 800, Jena, Germany) (Nair et al., 2016).

### Cutaneous Mouse Infection Model

Seven-week-old female mice CD-1 were purchased from Hunan Slake Jingda Experimental Animal, Co., Ltd. (Hunan, China). They weighed approximately 25 ± 3 g at the time of the experiments.

The high bacterial load abscess infection model was performed as defined earlier with slight adaptations (Pletzer et al., 2018). Before the injection, bacterial cells were rinsed resuspended in 1 × PBS. An injection of bacterial suspension was given to the dorsum to achieve the concentrations to generate reproducible abscesses and bacterial counts: *S. aureus*, 1 × 10^8^ CFU/mice; and *E. faecalis*, 1 × 10^9^ CFU/mice. Antimicrobial administration was given directly into the subcutaneous space of the infected area at 1 h post-infection. The development of the infection was observed every day. Abscesses were determined on day 2 using a caliper. Skin abscesses were removed (comprising all accrued pus) and regimented in sterile PBS by an automatic tissue homogenizer (Servicebio KZ-II, Wuhan, China). Bacterial counts were quantified by serial dilution. For histopathological analyses, hematoxylin and eosin (H&E) staining was performed.

### Statistical Analysis

Statistical evaluations were performed using GraphPad Prism 7.0. Checkerboard methods were performed at least in biological duplicates, and other experiments were performed in triplicate.

## Results

### Determination of the Susceptibility of Planktonic Cells

The MICs of AF and VAN against type strains and clinical isolates of *S. aureus* (MSSA/MRSA), *S. epidermidis*, and *E. faecalis* were 0.125–2 mg/L. The MBCs against *S. aureus* and *S. epidermidis* were 0.5–4 and 1–8 mg/L for AF and VAN, respectively. And the susceptibility of AF against MRSA and MSSA strains showed no difference. However, the MBCs of *E. faecalis* were > 32 mg/L for both AF and VAN (Table 1). In all, the strains we tested were more sensitive to the AF treatment than the VAN treatment.

**TABLE 1:** Antimicrobial susceptibility testing of AF and VAN toward bacterial strains (mg/L).

**Organism**	**AF**		**VAN**	
	**MIC**	**MBC**	**MIC**	**MBC**
***S. aureus***				
ATCC 29213	0.25	4	1	8
ATCC 25923^b^	0.25	2	0.5	1
ATCC 43300^a^	0.25	2	1	1
Newman	0.125	4	2	4
LZB1^b^	0.5	4	1	2
RJ-2	0.125	2	1	8
SA1401	0.25	1	1	1
SA1414^b^	0.5	2	1	2
SA1418^a^	0.5	2	1	2
SA1419	0.25	2	1	4
SA1422^a^	0.25	1	1	2
SA1423	0.5	1	2	2
SA1427^a^	0.25	2	1	1
SA1435^a,b^	0.25	1	2	4
***E. faecalis***				
ATCC 29212^b^	0.25	>32	2	>32
EF1401	0.25	>32	1	>32
EF1402	0.25	>32	1	>32
EF1403	0.5	32	1	>32
EF1405^b^	0.5	>32	2	>32
EF1407^b^	0.5	>32	2	>32
EF1410	0.5	>32	2	>32
EF1411^b^	0.25	>32	1	>32
EF1412	0.5	>32	1	>32
EFF01	0.5	>32	1	>32
EFF09^b^	0.5	>32	1	>32
EFF11	0.5	>32	1	>32
***S. epidermidis***				
RP62A^b^	0.125	2	2	8
ATCC 12228	0.125	4	1	4
SE1801	0.125	1	2	2
SE1802	0.125	1	2	2
SE1803	0.125	0.5	2	2
SE1804	0.125	0.5	1	1
SE1805	0.125	1	2	4
SE1806	0.125	2	1	1
SE1807	0.125	1	2	2
SE1808	0.125	1	2	2
SE1809^b^	0.125	0.5	2	4
SE1810^b^	0.125	1	2	4

*^*a*^Methicillin resistant *Staphylococcus aureus* (MRSA). ^*b*^Biofilm formation positive strains determined by CV staining method.*

### Synergistic Effect Between AF and Antibiotics Against Planktonic Cells

The synergistic effects of AF were investigated with some conventional systemic antibiotics (antibiotics with MIC values greater than 256 mg/L were excluded). The results of the combination screening assay are presented in Table 2. Synergistic interactions between AF and FOF (FICI = 0.375) or LZD (FICI = 0.375) were observed against *S. aureus* LZB1. For *E. faecalis* ATCC29212, synergistic interactions were observed between AF and CHL (FICI = 0.375). But no interactions between AF and antibiotics were observed against *S. epidermidis* RP62A (FICI > 0.5). Combinations with the lowest FICI values were selected for other representative strains. As shown in Table 3, the combination of AF and CHL still showed synergistic effect against the *E. faecalis* clinical isolates; combinations of AF + LZD/FOF still showed synergistic effects against *S. aureus* ATCC43300 (MRSA) and most of the clinical isolates, except for strain SA1435 which showed no interaction between AF and FOF with FICI of 0.5.

**TABLE 2:** The combinational antibacterial activities of AF and different antibiotics.

**Organism**	**Agent**	**MIC (μg/mL)**		**MIC_In combination_/MIC_singly_**	**FICI**	**Outcome**
		**Singly**	**In combination**			
*S. aureus* LZB1	FOF	0.5	0.125	0.25	0.375	Synergy
	AF	0.5	0.0625	0.125		
	LZD	4	1	0.25	0.375	Synergy
	AF	0.5	0.0625	0.125		
	CLI Hydro	0.125	0.0625	0.5	1	No interaction
	AF	0.5	0.25	0.5		
	GEN	4	2	0.5	1	No interaction
	AF	0.5	0.25	0.5		
	LVX	0.25	0.25	1	2	No interaction
	AF	0.5	0.5	1		
	VAN	1	0.5	0.5	1	No interaction
	AF	0.5	0.25	0.5		
*E. faecalis* ATCC29212	LZD	2	0.5	0.25	0.75	No interaction
	AF	1	0.5	0.5		
	CHL	4	0.5	0.125	0.375	Synergy
	AF	1	0.25	0.25		
	TET	16	4	0.25	0.5	No interaction
	AF	1	0.25	0.25		
	TEC	0.25	0.031	0.125	0.625	No interaction
	AF	1	0.5	0.5		
	CIP	0.5	0.125	0.25	0.75	No interaction
	AF	1	0.5	0.5		
	LVX	1	0.25	0.25	0.5	No interaction
	AF	1	0.25	0.25		
	CLI Hydro	32	1	0.031	0.531	No interaction
	AF	1	0.5	0.5		
	VAN	2	0.5	0.25	0.75	No interaction
	AF	1	0.5	0.5		
*S. epidermidis* RP62A	FOF	1	1	1	2	No interaction
	AF	0.25	0.25	1		
	CIP	0.25	0.125	0.5	1	No interaction
	AF	0.25	0.125	0.5		
	TEC	2	2	1	2	No interaction
	AF	0.25	0.25	1		
	LZD	2	1	0.5	0.625	No interaction
	AF	0.25	0.0313	0.125		
	CHL	16	8	0.5	0.516	No interaction
	AF	0.25	0.004	0.015		
	LVX	0.25	0.25	1	2	No interaction
	AF	0.25	0.25	1		
	TET	0.25	0.25	1	2	No interaction
	AF	0.25	0.25	1		

**TABLE 3:** The antibacterial activity and combined effects of AF and selected antibiotics alone or in combination against MRSA and other clinical isolates.

**Organism**	**Agent**	**MIC (mg/L)**		**MIC_In combination_/MIC_singly_**	**FICI**	**Outcome**
		**Singly**	**In combination**			
***S. aureus***						
ATCC43300 (MRSA)	LZD	2	0.25	0.125	0.375	Synergy
	AF	0.25	0.0313	0.25		
SA1435 (MRSA)	LZD	4	0.5	0.125	0.375	Synergy
	AF	0.5	0.125	0.25		
ATCC43300 (MRSA)	FOF	8	2	0.25	0.375	Synergy
	AF	0.25	0.0313	0.125		
SA1435 (MRSA)	FOF	32	8	0.25	0.5	No interaction
	AF	0.5	0.125	0.25		
***E. faecalis***						
EF1402	CHL	8	1	0.125	0.375	Synergy
	AF	0.25	0.0625	0.25		
EF1403	CHL	16	0.25	0.016	0.266	Synergy
	AF	0.5	0.125	0.25		

### Determination of the Susceptibility of Biofilms

**FIGURE 1:**
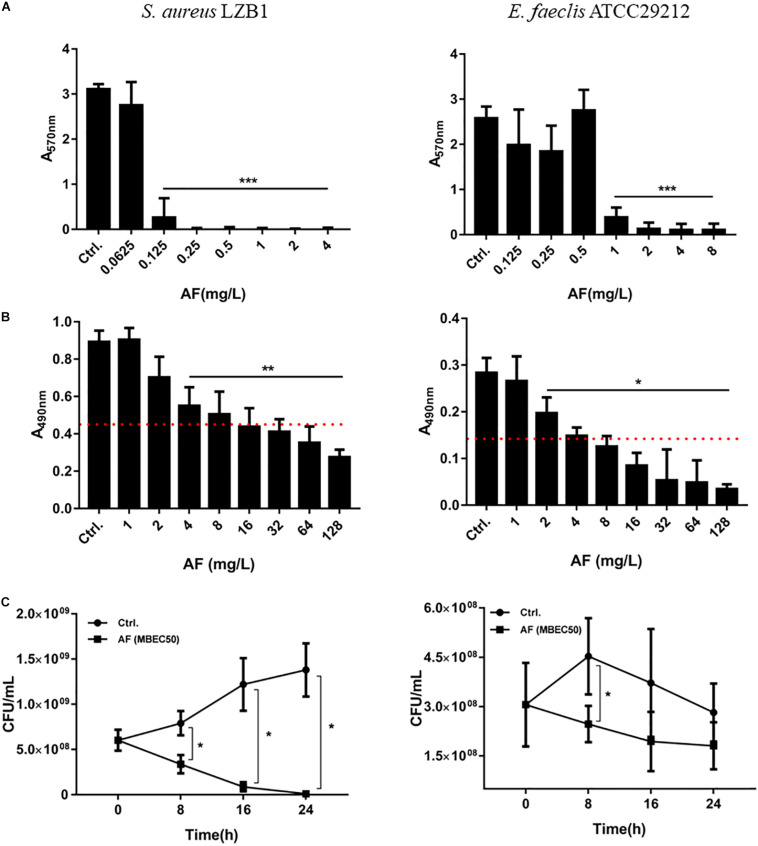
Antibiofilm effects of AF on *Staphylococcus aureus* LZB1 and *Enterococcus faecalis* ATCC29212. **(A)** Biofilm inhibitory effect determination by CV staining. Overnight cultures of strains were diluted with AF to the designated concentrations. After 24 h incubation, planktonic cells were removed and stained with 0.25% CV. **(B)** Biofilm eradication by AF detected by XTT staining. Biofilms grown for 24 h were treated with AF at the designated concentrations. After incubation, planktonic cells were removed and stained with a solution of XTT/PMS. The red dashed line indicates 50% of the biofilm biomass of the control group. **(C)** Live cell counts. Twenty-four hour biofilms were treated with AF at a concentration of MBEC50 for 24 h, and serial dilutions and plate counts were performed to determine the live cells in the biofilms (^∗^*p* < 0.05; ^∗∗^*p* < 0.01; ^∗∗∗^*p* < 0.001).

*Staphylococcus aureus* LZB1 and *E. faecalis* ATCC29212 were selected to test the antibiofilm activities of AF due to their strong biofilm formation abilities (Kart et al., 2017). AF showed strong biofilm inhibitory effects against *S. aureus*, and *E. faecalis* at concentrations of 0.125 and 1 mg/L (*p* < 0.05), respectively, in a dose-dependent manner (Figure 1A), which were very close to its MICs, indicating that the biofilm inhibitory effect of AF could be mainly due to its bacteriostatic or bactericidal activity by targeting thiol-redox homeostasis (Harbut et al., 2015). Because biofilm formation strongly increased the antimicrobial resistance to AF, the lowest concentrations needed to eradicate preformed biofilms were up to 4 and 2 mg/L for *S. aureus* and *E. faecalis*, respectively (Figure 1B). MBEC50 was selected to detect the time kill efficacy of AF against biofilms. AF showed significant biofilm killing activity against these strains in a time-dependent manner. Compared to the control group, AF reduced the live biofilm cells of *S. aureus* from (1.38 ± 0.29) × 10^9^ CFU/ml to (1.13 ± 0.90) × 10^7^ CFU/ml (*p* < 0.001). Although statistical significance was only observed at 8 h after treatment (*p* < 0.05), AF killed *E. faecalis* ATCC29212 biofilm cells throughout the 24 h period (Figure 1C). In addition, AF could also effectively eradicate clinical isolates with low MBEC50 values (Supplementary Table S2).

### Synergistic Effect Between AF and Antibiotics Against Biofilms

**FIGURE 2:**
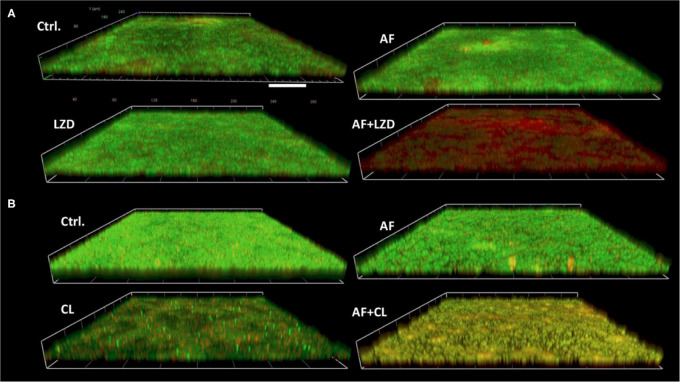
Demonstrative CLSM images of biofilm eradication by AF mono-/combination treatment. Biofilms were performed on glass cover slides and then treated with AF and antibiotics alone and/or in combination for 24 h. The cover slides were stained with the fluorescent dye mixture of SYTO9 (live cells, green) and PI (dead cells, red). **(A)** *S. aureus* LZB1, AF 8 mg/L, LZD 16 mg/L. **(B)** *E. faecalis* ATCC29212, AF 2 mg/L, CHL 8 mg/L. Scale bar: 40 μm.

Antibiotics that showed a synergistic effect on planktonic cells were tested against preformed biofilms in combination with AF (Table 4). AF significantly promoted the antibiofilm efficacy of CHL against *E. faecalis* ATCC29212 (4- and 8-fold decrease of MBEC50 for CHL and AF, respectively). Meanwhile, AF also increased the antibiofilm activity of LZD against *S. aureus* LZB1 and exhibited a 2- and > 8-fold decrease of MBEC50 for AF and LZD, respectively, but showed no interaction with FOF (Supplementary Figure S1). Similar observations were made by visualization of AF and/or antibiotic-treated biofilms by CLSM, when AF used in combination with FOF (*S. aureus* LZB1, Figure 2A) or CHL (*E. faecalis* ATCC29212, Figure 2B), the live cells in the biofilms were significantly reduced, although some intact patches of biofilm could still be visualized.

**TABLE 4:** Activity of AF in combination with conventional antibiotic against preformed biofilms (mg/L).

**Organism**	**Agent**	**Singly MBEC50**	**In combination MBEC50**	**Fold decrease of MBEC50 in combination**
*S. aureus* LZB1	AF	16	8	2
	LZD	>128	16	>8
	AF	16	16	–
	FOF	>64	>64	–
*E. faecalis* ATCC 29212	AF	8	2	4
	CHL	64	8	8

**FIGURE 3:**
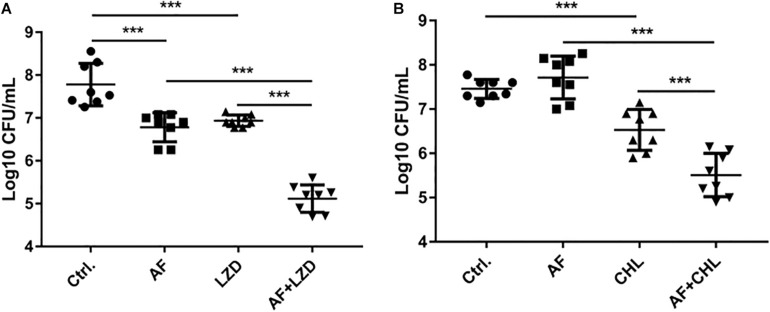
Antibiofilm activity of AF alone/in combination with antibiotics on preformed isolated biofilms on catheters. Biofilms on the surfaces of catheters were treated with AF, LZD, or CHL as described in Section “Materials and Methods.” Viable cells remaining on the catheter surface were counted by serial dilution method. **(A)** *S. aureus* LZB1, AF 8 mg/L, LZD 16 mg/L. **(B)** *E. faecalis* ATCC29212, AF 2 mg/L, CHL 8 mg/L (^∗^*p* < 0.05; ^∗∗^*p* < 0.01; ^∗∗∗^*p* < 0.001).

To simulate the *in vivo* conditions for biofilm formation in device-associated infections, we allowed strains to form biofilms on the surfaces of catheters. Treatment of biofilms with AF and in combination with antibiotics led to their synergistic eradication (Figure 3). A single dose of AF or antibiotics only showed moderate antibiofilm effects; however, combination treatment led to a 4.96- and 1.95-log reduction in CFUs for *S. aureus* (AF + LZD, Figure 3A) and *E. faecalis* (AF + CHL, Figure 3B), respectively, confirming that AF possesses antibiotic-promoting activity against preformed biofilms on catheters.

### Therapeutic Efficacy of AF Combined With Conventional Antibiotics *in vivo*

**FIGURE 4:**
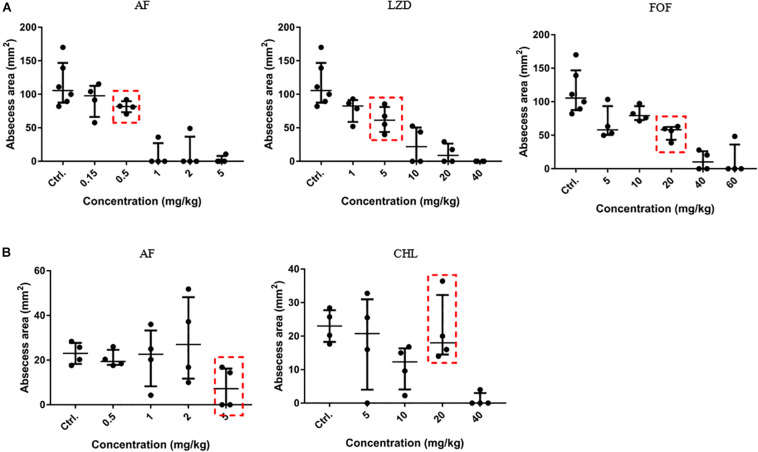
Dose-dependent bactericidal effect of antimicrobials against cocci *in vivo.* **(A)** AF, LZD, and FOF inhibited abscess formation of *S. aureus* LZB1 in a dose-dependent manner (the three groups share the same control group). AF and CHL inhibited abscess formation of *E. faecalis* ATCC29212 **(B)**. The abscess model was performed by subcutaneous injection of *S. aureus* LZB1 (1 × 10^8^ CFU/mouse), and *E. faecalis* ATCC29212 (1 × 10^9^ CFU/mouse). One hour later, 100 μL antimicrobials at the designated concentrations were directly subcutaneously injected. The abscess size was observed after 2 days.

To optimize the treatment strategy, antimicrobials were chosen based on their moderate *in vivo* pharmacodynamics (Figure 4), and the concentrations used in the present study were equal or less than those empirically tested *in vivo* (CHL, 10 mg/kg; LZD, 60 mg/kg; and FOF, 100 mg/kg) (Shibl, 1982; Guo et al., 2013; Zykov et al., 2018) to determine an appropriate concentration that reduces abscess sizes just enough to observe the synergy between the AF and the antibiotics (Figure 4, red dashed line). A significant reduction in the mean bacterial load was observed for each combined treatment condition compared with the control (receiving DMSO or Tween-80) or single dose group.

**FIGURE 5:**
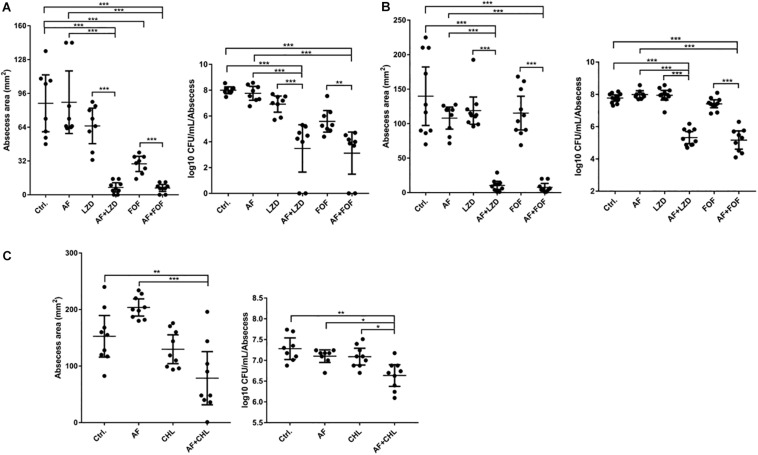
AF and antibiotic mono- and combinatorial therapy in a murine cutaneous abscess model using female CD-1 mice. Bacterial strains were injected subcutaneously and treated 1 h post-infection with either saline/DMSO/Tween-80 (control), AF, antibiotics, or antibiotic-AF combinations. AF concentrations for all conditions were as follows: 0.5 mg/kg for *S. aureus* LZB1 **(A)** and ATCC43300 (MRSA) **(B)**, and 5 mg/kg for *E. faecalis* ATCC29212 **(C)**. Infected and inflamed tissue was measured 2 days post-infection and pus-containing abscess lumps were excised to determine CFU. Abscess sizes are shown in the left panel and counted CFU/ml/abscess data is expressed in the right panel. **(A,B)** *S. aureus* LZB1, LZD 5 mg/kg, and FOF 20 mg/kg, respectively. **(C)** *E. faecalis* ATCC29212, CHL 20 mg/kg. All experiments were performed at least three times with 2–4 mice/group. Mean total abscess size (mm^2^) ± the standard error of the mean (SEM). Statistical analysis was performed using one-way ANOVA, Kruskal–Wallis test with Dunn’s correction (two-sided). The asterisk indicates significant differences between two groups (^∗^*p* < 0.05; ^∗∗^*p* < 0.01; ^∗∗∗^*p* < 0.001).

**FIGURE 6:**
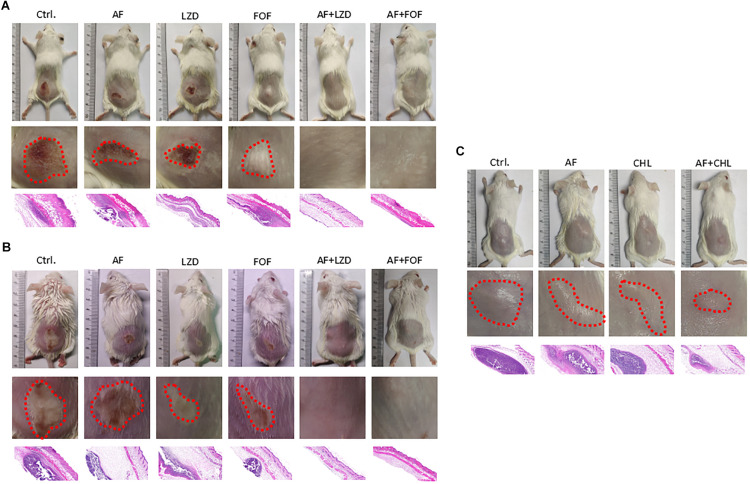
Representative photographs of the cutaneous abscess in the presence/absence of AF and antibiotic mono- or combinatorial therapy. An entire dorsal back (upper panels) and close-up images of the abscess (red circle, middle panel) and representative histological results (H&E stain, 50 ×, down panels) are shown at 2 days after therapy for **(A)** *S. aureus* LZB1, **(B)** *S. aureus* ATCC43300, and **(C)** *E. faecalis* ATCC29212.

Except for FOF, which reduced the abscess area of *S. aureus* LZB1 57.38 mm^2^, single use of AF (0.5 mg/kg), LZD (5 mg/kg) or FOF (20 mg/kg) showed no statistical significance in reducing abscess area or bacterial loads of *S. aureus* LZB1 (MSSA) (Figure 5A) and ATCC43300 (MRSA) (Figure 5B) infections; however, AF combined with LZD significantly decreased the abscess area and reduced the bacterial load for 4.51- (*S. aureus* LZB1, *p* < 0.01) and 2.45-fold log10 (*S. aureus* ATCC43300, *p* < 0.001). Similarly, AF or CHL could not inhibit the abscess growth of *E. faecalis* ATCC29212 individually, but when combined, the area of abscess was reduced by 74.14 mm^2^ (*p* < 0.01). Single use of AF or CHL had no impact on bacterial load; however, combined therapy reduced the bacterial load by 0.61-fold log10 (*p* < 0.01) (Figure 5C). For *in vivo* observations, the abscesses caused by *S. aureus* were more obvious than those caused by *E. faecalis*. The ulcers were formed when infected with *S. aureus* LZB1 or ATCC43300 (Figures 6A,B); however, infection with a high load of *E. faecalis* ATCC29212 (Figure 6C) only caused subcutaneous lumps. In accordance with the *in vitro* observations, the representative pictures of abscesses and histological examinations showed that single use of AF, LZD, or FOF showed no/moderate activity against infections caused by *S. aureus* LZB1 (Figure 6A) or ATCC43300 (Figure 6B), and extensive inflammation with leukocyte infiltration emerged; however, drug combination (AF + LZD or AF + FOF) significantly reduced the size and inflammation of the abscesses, which even eventually disappeared. The single use of AF or CHL had no influence on the abscesses caused by *E. faecalis* ATCC29212, the drug combination significantly diminished the abscess size and inflammation infiltration (Figure 6C). These important observations highlight that antimicrobial monotherapies are often ineffective when bacteria form high-density infections for *S. aureus* and *E. faecalis*. In addition, drug combinations could significantly improve the efficacy.

## Discussion

In the present study, the antimicrobial activity of AF was assessed against a panel of type strains and clinical isolates of *Staphylococcus* spp. and *E. faecalis.* In accordance with the previous studies reported by Cassetta et al. (2014), Harbut et al. (2015), Fuchs et al. (2016), the MICs for *S. epidermidis*, *S. aureus* (including MSSA and MRSA), and *E. faecalis* were 0.125–0.5 mg/L, which showed more susceptibility than with VAN treatment, with MICs ranging from 0.5 to 2 mg/L.

Drug combination is a promising way to improve the efficacy of drugs and reduce side effects and cytotoxicity. In our study, highly synergistic interactions between AF and CHL were observed against *E. faecalis*. CHL is a broad-spectrum antibiotic against many gram-positive/negative bacteria (Civljak et al., 2014). However, CHL is an old antimicrobial agent that is rarely used today mainly due to its most significant adverse effect of dose-related bone marrow suppression, according a meta-analysis by Eliakim-Raz et al. (2015), CHL is as safe a treatment alternatives as short antibiotic courses. In this way, drug combination could significantly diminish the dose required but achieve better antimicrobial efficacy, so that AF combined with CHL could be a better choice than CHL used alone in clinical therapy. Moreover, *E. faecalis* has shown many different metabolic responses from anaerobic to aerobic circumstances; these main metabolic cascades are related to the response to nutrients and may change the susceptibility of this bacterium to bactericidal drugs (Portela et al., 2014). However, even in the anaerobic condition, AF still showed a highly synergistic effect with CHL (Supplementary Table S1).

Highly synergistic interactions between AF and LZD/FOF were also observed against MSSA or MRSA strains of *S. aureus*. LZD has a wide spectrum of action against the mainstream of common gram-positive cocci. However, due to the development of resistance to antibiotics as well as their unwanted side effects, combination therapy has evolved as an imperative novel treatment approach (Yang et al., 2018). FOF by itself has a bactericidal impact both *in vitro* as well as *in vivo*. Nevertheless, MRSA can easily develop tolerance, making utilization of FOF unattainable for medical situations (Roussos et al., 2009). FOF displays positive synergistic impacts on MRSA and its biofilms when utilized in combination with other antibiotics (Shi et al., 2014). Similarly, in our study, AF showed an excellent synergistic effect with FOF against MSSA and MRSA planktonic cells. In addition, different clinical isolates showed outcomes of different drug combinations, which indicates the importance of an *in vitro* synergistic test before clinical use.

Biofilms are easily formed on indwelling medical apparatus surfaces (Francolini and Donelli, 2010). During implantation of catheters, tissue damage might occur due to the buildup of platelets as well as fibrin at the suture site as well as on the devices. Microbial cells have enhanced capability to colonize these sites (Jamal et al., 2018). The formation of biofilms increases the antibiotic resistance and leads to persistent infections posing major healthcare challenges. AF showed modest biofilm inhibitory and eradicating effects against *Staphylococcus aureus* and *E. faecalis* both in type strains and clinical isolates with high values of MBEC70. Thus, our favorable outcomes of synergy among AF and antibiotics encouraged us to inspect the action of drug combinations against biofilms. AF combined with CHL showed synergistic antibiofilm effects for *E. faecalis* on cover slides or infusion catheters (Supplementary Figure S1), AF significantly improved the antibiofilm effects of LZD against *S. aureus*. As numerous antibiotics have diverse antibacterial mechanisms and several bacteria have diverse resistance mechanisms, to entirely eliminate the whole biofilm-bacteria is a difficult challenge. Combination therapy comprising two or more antibiotics with diverse bactericidal mechanisms could synergistically eliminate biofilms (Simoes, 2011).

A high bacterial load-containing abscess model has rarely been studied for AF efficacy evaluation. In our *in vivo* subcutaneous abscess model study, single use of antimicrobials showed an extremely modest effect on abscess area or bacterial load. However, AF combined with LZD or FOF synergistically inhibited abscess and inflammation formation and reduced the bacterial load for both MSSA and MRSA strains. The safety of AF in *in vivo* animal studies and clinical use is well-documented. AF is widely used in clinical settings for long-term treatment at the daily dosage at 6 mg/day, and a average blood concentration of 3.5 μM (∼2.38 μg/ml, which is far beyond the value of MICs) is reached in 12 weeks. Besides, the effectiveness and safety of AF at an dose of 12 mg/day is under Phase II clinical trial (Harbut et al., 2015). As reported by Aguinagalde et al. (2015), the dosage of AF used for murine model even reach to 10 mg/kg due to its safe toxicity profile and well-known pharmacokinetic/pharmacodynamic characteristics. Similarly, the safety of antibiotics of FOF and LZD is well-studied and documented. As reported by Pachón-Ibáñez et al. (2011) and Guo et al. (2013), the dosages of FOF and LZD used are reached to 100 and 60 mg/kg in murine models, respectively. And the FOF and LZD used in our study are only 20 and 5 mg/kg, respectively. Therefore, the inflammation caused in our animal models is not caused by the antimicrobials we used. In all, the combination therapy of AF plus LZD/FOF might be an effective option for treating patients with *S. aureus*-related subcutaneous abscess infection. Similarly, AF combined with CHL also showed synergistic antibacterial effects on *E. faecalis* abscesses and partially reduced inflammation formation.

## Conclusion

The present study provides a valuable effect of antimicrobial combination therapy against cocci in subcutaneous abscess infections. This type of synergistic combination of two medications is likely preferred in clinical situations. The rationality of the outcomes should be validated by future clinical trials.

## Data Availability Statement

All datasets generated for this study are included in the article/Supplementary Material.

## Ethics Statement

Ethical approval was obtained from the Animal Ethics Committee (certificate number 2017-S139), the Third Xiangya Hospital, Central South University, China.

## Author Contributions

PS and YW designed and performed the experiments and wrote the manuscript. PS, YW, and LZ performed the experiments and data collection. SL, YL, LC, and ZL performed the experiments and revised the manuscript. All authors read and approved the final manuscript.

## Conflict of Interest

The authors declare that the research was conducted in the absence of any commercial or financial relationships that could be construed as a potential conflict of interest.

## Supplementary Material

FIGURE S1 Representative CLSM images of biofilm eradication by AF and FOF mono-/combination treatment. *S. aureus* LZB1 biofilms on the surfaces of cover slides were treated with AF (16 mg/L) and/or FOF (128 mg/L) as described in Section “Materials and Methods,” the stained with the SYTO9/PI fluorescent dye mixture.

TABLE S1 The combinational antibacterial activities of AF and different antibiotics against *E. faecalis* ATCC29212 in anaerobic conditions.

TABLE S2 Biofilm eradication activities of AF against other type and clinical strains (mg/L).
