# Corrigendum: Synergistic Microbicidal Effect of Auranofin and Antibiotics Against Planktonic and Biofilm-Encased *S. aureus* and *E. faecalis*

**DOI:** 10.3389/fmicb.2021.694670

**Published:** 2021-06-04

**Authors:** Pengfei She, Linying Zhou, Shijia Li, Yiqing Liu, Lanlan Xu, Lihua Chen, Zhen Luo, Yong Wu

**Affiliations:** Department of Clinical Laboratory, The Third Xiangya Hospital of Central South University, Changsha, China

**Keywords:** auranofin, biofilm, combination therapy, subcutaneous abscess model, *Staphylococcus aureus*, *Enterococcus faecalis*

**Error in Figure**

In the original article, there was a mistake in Figure 2A as published. **The original image of the control group was incorrect**. The corrected Figure 2 appears below.

**Figure 2:**
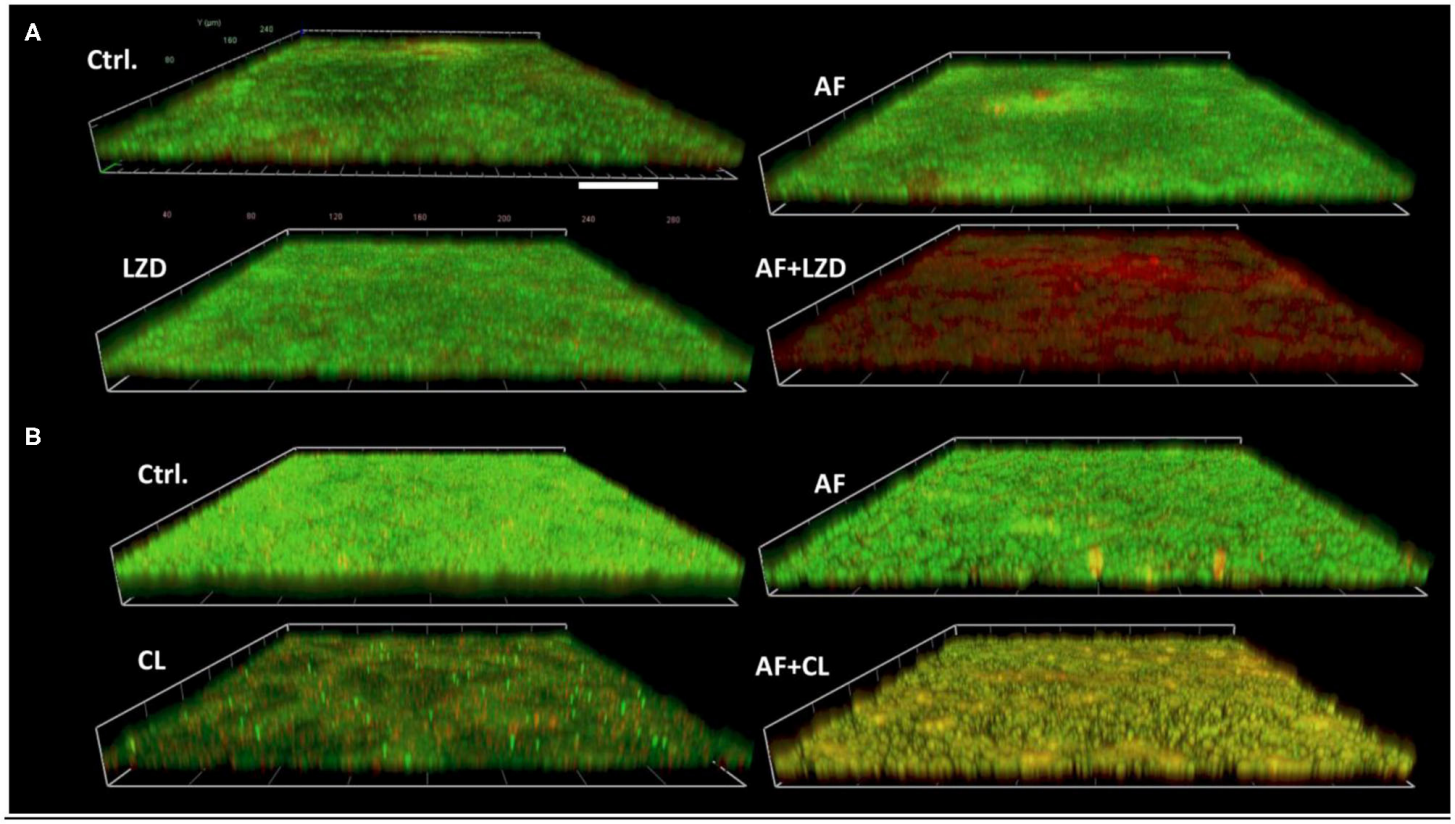
Demonstrative CLSM images of biofilm eradication by AF mono-/combination treatment. Biofilms were performed on glass cover slides and then treated with AF and antibiotics alone and/or in combination for 24 h. The cover slides were stained with the fluorescent dye mixture of SYTO9 (live cells, green) and PI (dead cells, red). **(A)** *S. aureus* LZB1, AF 8 mg/L, LZD 16 mg/L. **(B)** *E. faecalis* ATCC29212, AF 2 mg/L, CHL 8 mg/L. Scale bar: 40 μm.

The authors apologize for this error and state that this does not change the scientific conclusions of the article in any way. The original article has been updated.

