# Supplementary figures and images for: Synergistic Microbicidal Effect of Auranofin and Antibiotics Against Planktonic and Biofilm-Encased S. aureus and E. faecalis
Source: Front Microbiol. 2019 Oct 24;10:2453. doi: 10.3389/fmicb.2019.02453 (PMC6821689; doi:10.3389/fmicb.2019.02453)

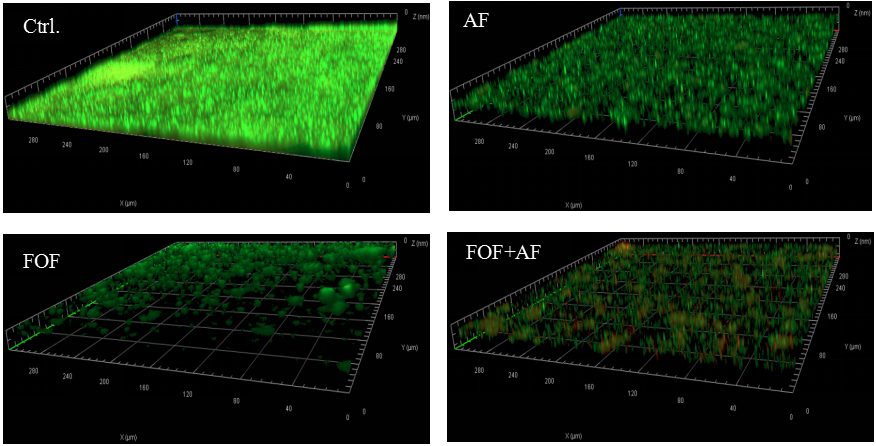

Supplement: FIGURE S1 — Representative CLSM images of biofilm eradication by AF and FOF mono-/combination treatment. S. aureus LZB1 biofilms on the surfaces of cover slides were treated with AF (16 mg/L) and/or FOF (128 mg/L) as described in Section “Materials and Methods,” the stained with the SYTO9/PI fluorescent dye mixture. [file Image_1.TIF]
