# Supplementary material for: Synergistic Microbicidal Effect of Auranofin and Antibiotics Against Planktonic and Biofilm-Encased S. aureus and E. faecalis
Source: Front Microbiol. 2019 Oct 24;10:2453. doi: 10.3389/fmicb.2019.02453 (PMC6821689; doi:10.3389/fmicb.2019.02453)
Supplement: TABLE S1 — The combinational antibacterial activities of AF and different antibiotics against E. faecalis ATCC29212 in anaerobic conditions. [file Table_1.doc]

TABLE S1. The combinational antibacterial activities of AF and different antibiotics against *E.faecalis* ATCC29212 in anaerobic conditions.

| Agent | MIC(μg/mL) | | MIC_In combination_/MIC_singly_ | FICI | Outcome |
| --- | --- | --- | --- | --- | --- |
|  | Singly | In combination |  |  |  |

| CHL | 8 | 1 | 0.125 | 0.375 | Synergy |
| --- | --- | --- | --- | --- | --- |
| AF | 2 | 0.5 | 0.25 |
| TET | 16 | 0.5 | 0.031 | 0.531 | No interaction |
| AF | 2 | 1 | 0.5 |
| TEC | 0.25 | 0.0625 | 0.25 | 0.5 | No interaction |
| AF | 2 | 0.5 | 0.25 |
| CIP | 1 | 0.031 | 0.031 | 0.531 | No interaction |
| AF | 2 | 1 | 0.5 |
| CLI Hydro | 32 | 16 | 0.5 | 1 | No interaction |
| AF | 2 | 1 | 0.5 |
| LVX | 0.5 | 0.5 | 1 | 2 | No interaction |
| AF | 2 | 2 | 1 |
| VAN | 2 | 2 | 1 | 2 | No interaction |
| AF | 2 | 2 | 1 |
| LZD | 2 | 0.5 | 0.25 | 0.5 | No interaction |
| AF | 2 | 0.5 | 0.25 |
