# Supplementary material for: Synergistic Microbicidal Effect of Auranofin and Antibiotics Against Planktonic and Biofilm-Encased S. aureus and E. faecalis
Source: Front Microbiol. 2019 Oct 24;10:2453. doi: 10.3389/fmicb.2019.02453 (PMC6821689; doi:10.3389/fmicb.2019.02453)
Supplement: TABLE S2 — Biofilm eradication activities of AF against other type and clinical strains (mg/L). [file Table_2.DOC]

**TABLE S2**. Biofilm eradication activities of AF against other type and clinical strains (mg/L).

| Strains | MBEC30 | MBEC50 | MBEC70 |
| --- | --- | --- | --- |
| ***S.aureus*** | 4  8  0.25  8  2  8 | 16  64  4  8  16  64 |  |
| LZB1 |  |  | >128 |
| SA1414 |  |  | 128 |
| SA1435 |  |  | 32 |
| ***E.faecalis*** |  |  |  |
| ATCC 29212 |  |  | 32 |
| EF1405 |  |  | 128 |
| EF1411 |  |  | >128 |
